# Mixed-Effects Modelling of Scale Growth Profiles Predicts the Occurrence of Early and Late Fish Migrants

**DOI:** 10.1371/journal.pone.0061744

**Published:** 2013-04-16

**Authors:** Francisco Marco-Rius, Pablo Caballero, Paloma Morán, Carlos Garcia de Leaniz

**Affiliations:** 1 Departamento de Bioquímica, Genética e Inmunología, Universidad de Vigo, Vigo, Spain; 2 Consellería de Medio Rural, Servizo de Conservación da Natureza, Xunta de Galicia, Pontevedra, Spain; 3 Department of BioSciences, Swansea University, Swansea, United Kingdom; Universitat de Barcelona, Spain

## Abstract

Fish growth is commonly used as a proxy for fitness but this is only valid if individual growth variation can be interpreted in relation to conspecifics' performance. Unfortunately, assessing individual variation in growth rates is problematic under natural conditions because subjects typically need to be marked, repeated measurements of body size are difficult to obtain in the field, and recaptures may be limited to a few time events which will generally vary among individuals. The analysis of consecutive growth rings (circuli) found on scales and other hard structures offers an alternative to mark and recapture for examining individual growth variation in fish and other aquatic vertebrates where growth rings can be visualized, but accounting for autocorrelations and seasonal growth stanzas has proved challenging. Here we show how mixed-effects modelling of scale growth increments (inter-circuli spacing) can be used to reconstruct the growth trajectories of sea trout (*Salmo trutta*) and correctly classify 89% of individuals into early or late seaward migrants (smolts). Early migrants grew faster than late migrants during their first year of life in freshwater in two natural populations, suggesting that migration into the sea was triggered by ontogenetic (intrinsic) drivers, rather than by competition with conspecifics. Our study highlights the profound effects that early growth can have on age at migration of a paradigmatic fish migrant and illustrates how the analysis of inter-circuli spacing can be used to reconstruct the detailed growth of individuals when these cannot be marked or are only caught once.

## Introduction

Body size is often the direct target of natural selection [1]–[3] and examining how different individuals grow can reveal much about how they respond to competition and adapt to environmental change [4], [5]. However, modelling animal growth has proved challenging because there is considerable heterogeneity among species and individuals, and because accounting for such diversity is inherently difficult at the analytical level [6]. For example, homoeothermic and poikilothermic organisms show markedly different constraints on evolution of body size [7], and hence on growth. Growth can also vary markedly throughout the lives of organisms, and regional-scale processes can affect individuals very differently depending on season [8], [9]. Individuals may cease feeding or augment food intake depending on temporal cues but environmental thresholds may vary markedly among individuals giving rise to divergent growth trajectories even among neighbouring conspecifics exposed to the same cues [10]–[12]. In addition, growth is intimately linked to many life history traits [13], and cannot be considered in isolation. For example, rate of growth has a pervasive effect on age at maturity in fishes [14]–[16], and on longevity in mammals [17].

Anadromous salmonids are good models to examine the fitness consequences of individual variation in growth because resident and migratory individuals commonly coexist and alternative reproductive tactics are often size-dependent [18], [19] reflecting considerable phenotypic plasticity [20], [21]. Thus, the choice to remain in freshwater or to migrate to sea is often determined by size and/or growth thresholds [22] and these may in turn depend on metabolic efficiency [23]. However, movement can be triggered by two very different and seemingly opposing conditions in relation to energy acquisition and individual performance. Poor growers may be forced to move in response to competition and low food acquisition [24], [25], while fast growers may move because they have increasingly high food demands, as high metabolic rates are difficult to maintain in freshwater [26]. Thus, although movement can be viewed as a generalized response to adversity [27], the underlying causes can be very different and will likely have different fitness consequences.

Individual growth is often estimated as the difference in body size between two or more arbitrarily chosen time events, but in most field studies not all marked individuals can be recaptured, or even sampled at the same times [28], making individual comparisons difficult. More commonly, researchers are limited to inferring growth from changes in the average size at successive ages or time events [29]–[31], often based on different individuals. Growth is then analyzed using models based in the Von Bertalanffy [32] approximation [33]–[35], but this ignores individual variation in growth trajectories [36] and assumes that age is accurately measured [37], something that is not always possible.

Here we used a method based on the analysis of the spacing between consecutive growth rings found in the scales (growth circuli) to reconstruct individual growth trajectories of migratory brown trout (sea trout, *Salmo trutta*) before they migrated to sea. We specifically compared the growth trajectories of individuals that migrated to sea after only one year in the river (early migrants) with those that delayed seaward migration for one or more additional years (late migrants). We hypothesized that early migrants would have grown faster than late migrants during their first year of life if migration was triggered by high food demands (i.e. intrinsic drivers), but would have grown more slowly if migration was the result of extrinsic constraints, for example competition resulting in low food acquisition.

## Methods

### Study populations

Migratory brown trout (sea trout) were caught in upstream traps or by angling in the rivers Ulla and Lerez (Galicia, NW Spain) during 1999–2010. The two populations differ in key physical and demographic parameters, including accessible length (Lerez = 25.1 Km, Ulla = 102.3 Km), watershed area (Lerez = 449.5 Km^2^, Ulla = 2803.6 Km^2^) and stream order (Lerez = 5, Ulla = 6). Mean angling catch of migratory trout during (used as a proxy for population size) was 101 individuals in the R. Lerez and 1,934 individuals in the R. Ulla. Sea trout smolts in the R. Ulla tend to be older (mean smolt age 2.2±0.45 yrs) and larger (mean smolt size 216±44 mm) than those in the R. Lerez (mean smolt age 2.1±0.58 yrs; mean smolt size 191±52 mm), while mean age at return shows the opposite pattern (R. Ulla, 3.15±0.84 yrs; R. Lerez, 3.51±0.78

### Scale analysis and reconstruction of growth profiles

Scales of 60 individuals per river and year were stored dry in paper envelopes, along with information on their body size (fork length, mm). Between three and five scales with a clear nucleus were selected per individual to minimize bias due to scale regeneration [38]. Acetate impressions were made with the aid of a pressure roller and the resulting impressions scanned at 23–50× magnifications (Minolta MS 6000) as in [39]. The software Image-J v. 1.4.1 [40] was employed to digitize the position of each growth ring (circulus) and to measure inter-circuli spacing with reference to a calibrated scale bar [41]. Freshwater and marine ages were determined based on the number of annuli [42], and the point of entry of smolts into the sea identified by a change from concave to convex circuli (i.e. the point where circuli open outwards on the posterior zone of the scale) and presence of broken growth rings [43].

### Reliability of scale analysis

A paired t-test was used to assess non-random deviations in scale radii between the original scales and their acetate impressions (n = 30) in order to quantify potential bias in scale measurements arising from pressure from the hand roller. To ascertain the precision of the scale analysis, we estimated the repeatability of the point of entry into the sea and of the end of the first freshwater growing season by measuring the scales of 30 individuals twice in a double blind fashion and calculating the intra-class correlation coefficient (α-Cronbach) as per [21]. The Pearson correlation coefficient was used to evaluate the strength of the association between scale radius and body size of fish in each river. The coefficients of variation (CV) were then examined to compare the precision of body size and scale measurements. Precision in scale measurements (0.01 mm; CV = 17.1%) was better than that of body size measurements (cm; CV = 19.2%), and the former was therefore preferred to examine growth variation among migratory trout. Thus, and in common with other recent studies [44]–[45], we did not convert scale measurements into body size estimates, as this would have merely introduced additional errors resulting from (1) low precision of body size measurements taken on live fish in the field, and (2) uncertainty about the precise nature of the function linking scale growth to body growth.

### Data analysis

In order to model individual variation in freshwater growth, we examined the spacing between consecutive growth circuli (inter-circuli spacing), the number of growth circuli deposited in the scales, and the growth of the scales (scale length) attained at the end of the first year. We only modelled variation in freshwater growth as we are interested in explaining drivers of seaward migration, and this allowed us to compare the common part of the scales of all migratory individuals, irrespective of the time they had spent at sea. We employed binary logistic regression to model the likelihood of early vs. late seaward migration as a function of growth during the first year of life, and assessed model fit by the log-likelihood ratio using SYSTAT 10.0. The first three circuli of the scale were not taken into account due to the possibility of missing growth rings during early life [44].

We used REML to model individual variation in inter-circuli spacing with the ‘nlme 3.1–86’ package [46] on the R 2.14 language [47]. A preliminary analysis of 20 randomly chosen individuals per river suggested that the inclusion of random effects was required to account for individual variation in slopes and intercepts (Figure S1, Supporting information). The inclusion of higher order polynomials allowed us to examine differences in the seasonal growth of one and two year old smolts, according to the following expression (Equation 1):
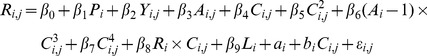
where *R* is the scale radius of individual *i* at circulus *j*, *P* is the population (R. Lerez, 0, R. Ulla 1), *Y* represents the different smolt years, *A* is the freshwater age, *C* is the scale circuli and *L* is the fork length of the fish returning from the sea. Random slopes (*b*) and intercepts (*a*) were assumed to be independent and normally distributed with zero means and variances σ^2^a and σ^2^b, respectively; errors ε_i_,_j_ were also assumed to be independent and normally distributed. We allowed for autocorrelation in inter-circuli spacing by considering an autoregressive (AR) model of order one in the autocorrelation structure, as this provided a better fit to the data than a model without correlated serial errors. The Bayesian Information Criteria (BIC) was used for model selection of fixed effects, mixed effects and autocorrelation structure of errors terms [48]. We employed the coefficient of variation (CV) to quantify individual variation in inter-circuli spacing at each circulus [21].

For the classical Von Bertalanffy growth model, a nonlinear least-squares (nls) regression was used to estimate the growth parameters (*L*∞, *k* and *t*_0_) from the scales, using all the scale circuli (Equation 2):
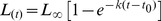
Individual growth curves were fitted to each fish and comparisons were made between fitted and observed values.

### Ethics Statement

We made use of scales samples collected routinely for ageing purposes by trained fisheries staff of the Regional Government of Galicia (Wildlife Service). Scales were collected from anaesthetized fish (clove oil) using a small fish knife and according to current Spanish Regulations, as described in a previous study [21]. After the fish had fully recovered from the anaesthesia (approx. 30 min), they were returned live to a point immediately upstream of the point of capture. No specific permits were required for scale collection, as these did not involve endangered or protected species and the work was carried out by government fishery officers under the supervision of one of the co-authors (PC), who is a government fish veterinarian.

## Results

### Reliability of scale analysis

The impression process produced no significant distortion of scale radius (*t*_29_ = 0.547, *P* = 0.465), indicating that acetate impressions gave an accurate, unbiased representation of scale size. Repeatabilities of scale size were high, both for smolt scale length (α-Cronbach = 0.879) and for scale size attained at the end of the first freshwater growing season (α-Cronbach = 0.898). Scale radius and fork length were positively correlated (*r* = 0.747, *P* = 0.001), and the relationship was not different between rivers (*F*_1,636_ = 0.542, *P* = 0.589) allowing us to examine individual variation in scale growth regardless of river identity.

### Effect of first year growth on age of seaward migration

**Figure 1:**
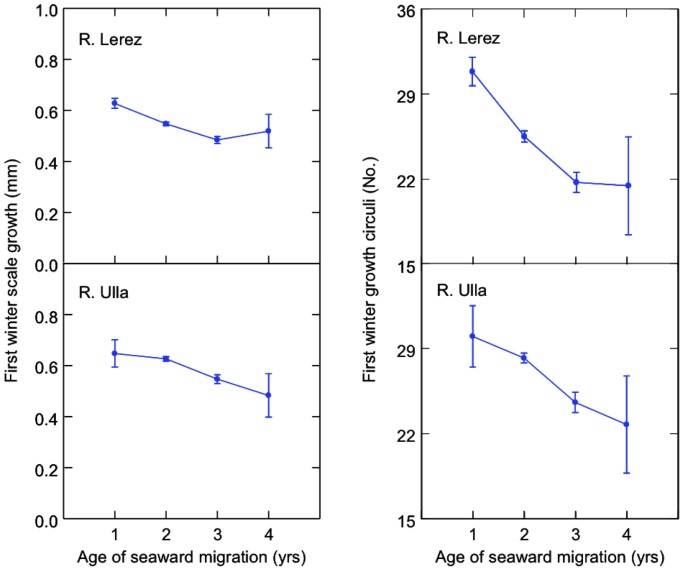
Marginal means (± SE) of (a) scale growth and (b) no. of growth circuli deposited during the first year in sea trout of different smolt ages (age of seaward migration).

**Figure 2:**
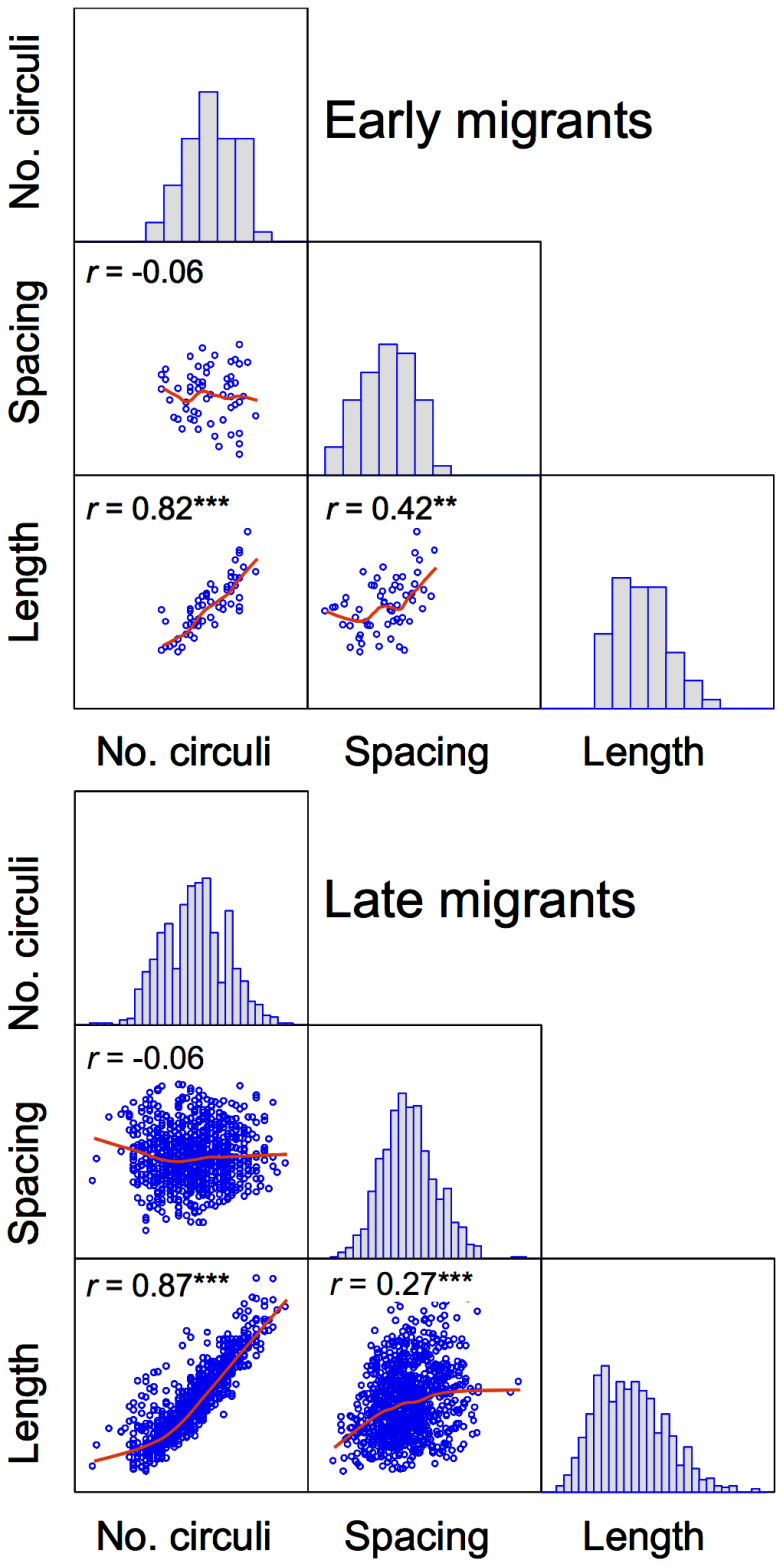
Scatterplot matrix showing relationships between scale growth parameters (no. of growth circuli, mean inter-ciculi spacing, and scale length during the first year in freshwater) of returning sea trout migrating to sea as early migrants (1 year old smolts) or late migrants (2–4 year old smolts). Each variable is compared against the other two and the relationship between pairs of variables is shown by a solid red line representing a locally weighted scatterplot smoothing (LOWESS) and by the strength of the Pearson correlation coefficient (***P<0.001; **P<0.01). Frequency histograms for each variable are shown along the diagonal.

The minimal adequate model that best explained age of seaward migration included scale growth (estimate = −4.5, SE = 0.96, *t* = −4.66, *P*<0.001) and mean inter-circuli spacing during the first year (estimate = 565.5, SE = 85.20, *t* = 6.64, *P*<0.001) as predictors. This provided a reasonably good fit to the data (McFadden rho^2^ = 0.138, *χ* ^2^ = 60.263, df = 2, *P*<0.001) and correctly classified 89.4% of fish into early and late migrants. The inclusion of other terms and their interactions did not improve model fit. In both rivers, early migrants (i.e. one year old smolts) were those that had attained higher rates of scale growth until their first winter (Fig. 1a; R. Lerez *F*_3,448_ = 13.123, *P*<0.001; R. Ulla *F*_3,486_ = 6.328, *P*<0.001). This was chiefly achieved by depositing more growth rings for a given scale length (Fig. 1b; R. Lerez *F*_3,448_ = 14.279, *P*<0.001; R. Ulla *F*_3,486_ = 5.906, *P* = 0.001). Thus, scale length and number of growth rings were positively correlated in both early and late migrants, but inter-circuli spacing and number of growth rings were not (Figure 2).

### Individual variation in freshwater growth

**Figure 3:**
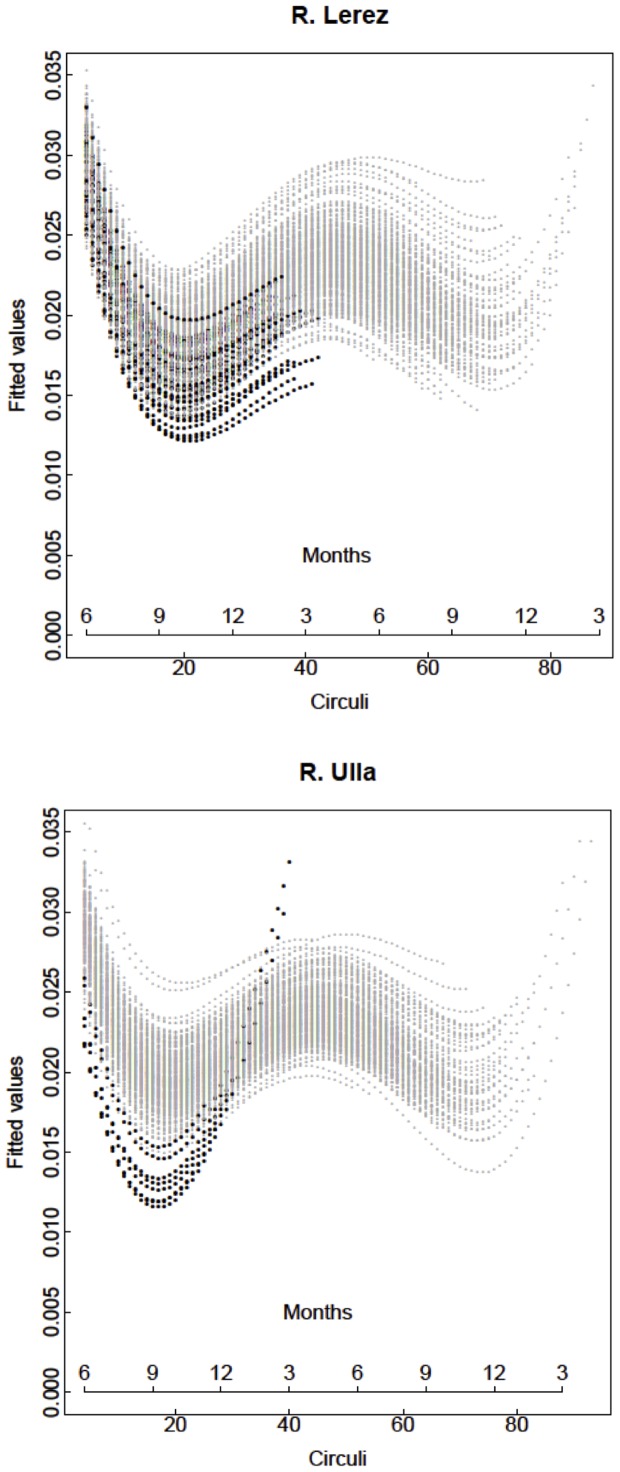
Fitted values of the mixed-effects model of inter-circuli spacing in the freshwater phase of migratory brown trout. Growth trajectories of one (•) and two-year old smolts (<$>\raster="rg1"<$>) are indicated. Correspondence between circuli number and calendar month is only approximate and is used to visualize the timing of seasonal growth stanzas.

The minimal adequate model for inter-circuli spacing in freshwater included an autocorrelation term as well as random slopes and intercepts (Table 1). Although random slopes and intercepts were relatively small, their inclusion significantly improved model fit, indicating that growth rates during early development, as well as initial size differences, differed significantly among individuals and affected subsequent growth. The positive and significant effect of fork length reflects the positive association found between body size and scale size, whereas the four polynomial terms of the model reflect the seasonality in inter-circuli spacing that was found to be necessary to include in order to capture seasonal growth stanzas (Figure 3), and which differed significantly between rivers. Thus, inter-circuli spacing for the River Ulla was significantly higher than for the River Lerez. In contrast, inter-circuli spacing did not vary significantly over the 11 years of study. Trout which emigrated to sea after only one year of growth in freshwater (early migrants) tended to have smaller than average inter-circuli spacing during the first months of life than those that delayed migration for one or two additional years. Such an effect was evident in both populations (Figure 1), and was largely the consequence of having deposited more growth rings for a given scale length. Analysis of temporal trends in CV indicates that variability in scale length increments increased until the first winter in both rivers (Figure S2), suggesting that initial differences in growth performance among individuals became amplified during early life. Model checks indicate a moderately good fit between observed and predicted values (Supporting information, Figure S3) and well behaved residuals. On the other hand, model fitting using individual Von Bertalanffy growth curves was very poor (*R* ^2^ = 0.04), and gave exponential or even negative growth for many fish (data not shown).

**Table 1:** Parameter estimates in mixed-effects modelling of inter-circuli spacing of sea trout in two study rivers.

Effects	Estimate	Std. Error	t-value	*P*-value
Fixed				
Intercept	0.0389	0.00071	54.54	<0.0001
FW age	−2.667 10^−4^	3.520 10^−4^	−0.75	0.449
River [Ulla]	0.0395	7.599 10^−4^	52.07	<0.0001
Circuli	−2.722 10^−3^	3.754 10^−5^	−72.52	<0.0001
Circuli^2^	1.222 10^−4^	1.815 10^−6^	67.33	<0.0001
Circuli^3^	−0.453 10^−7^	0.453 10^−8^	−49.72	<0.0001
Circuli^4^	0.190 10^−7^	0.024 10^−8^	53.01	<0.0001
FW age×Circuli^3^	1.001 10^−7^	1.401 10^−8^	7.22	<0.0001
River [Ulla]×Circuli	6.200 10^−7^	8.256 10^−6^	0.07	0.946
Fork length	3.480 10^−6^	1.234 10^−6^	2.82	0.004
Random (SD)				
Intercept	2.683 10^−3^			
Slope (Circuli)	8.706 10^−5^			
Residual	5.796 10^−3^			
Correlation structure				
corr	0.742			

Random effects are indicated by the standard deviation of slopes and intercepts.

## Discussion

How does one examine individual variation in growth rates when subjects cannot be marked or few are ever recaptured? We believe that the analysis of growth rings may provide an answer. Fish scales are routinely collected in fisheries for ageing, but it has long been recognised that the spacing between growth circuli (inter-circuli spacing) can also reveal much about the growth of individuals [49]. However, this information has traditionally proven difficult to analyze [50]; often researchers simply back-calculate body size at a given age, work on average size increments at particular times, or apply corrections to the degrees of freedom in an attempt to account for autocorrelations in inter-circuli spacing (e.g. [45]). We employed mixed effects modelling to compare inter-circuli spacing in the scales of migratory brown trout, and used this information to reconstruct juvenile growth trajectories in freshwater. To model seasonal changes in consecutive length increments, we included first to fourth order polynomial terms as a smoothing function of time [51], [52] and compared the results to those obtained by applying individual Von Bertalanffy equations.

Our results indicate that variation in early summer growth differed significantly among individuals (as evidence by the existence of random intercepts and slopes), and that individual differences in early growth became amplified over the first months of life (as evidenced by an increase in the coefficient of variation). Large individual differences in early growth have been documented in salmonids soon after emergence from the redd [53] and these appear to be closely related to dominance status [54], metabolic rate [55], and timing of hatching in relation to prior residency effects [56]. It is likely that the strength of density-dependent regulation during early life may determine the extent of individual variability in growth, so that fish which achieve high growth rates during their first year are able to maintain a size advantage later in life [57]. Small individuals within a cohort are more influenced by intra-specific competition than large ones [58]. In this sense, incorporating random effects in the model, allows a better insight into the nature of within-individual variation in growth patterns.

Egg size has been found to have a large effect on early growth and survival of salmonids [59], [60] and it is possible that the large individual differences revealed by our analysis might be related to variation in egg size (maternal effect; [61]) in addition to differences in early rearing [62]. In addition, we found significant differences in the freshwater growth of sea trout inhabiting two neighbouring rivers. This serves to highlight the large influence that, in addition to maternal effects [62], local-scale rearing conditions may have on juvenile salmonids during early life [53], [63]. The significant effect of body size in our model further suggests the growth experienced in freshwater has a positive effect on length at maturity. Marine survival of anadromous fish is thought to depend not just on growth attained at sea (e.g. [64]), but also on the size attained by juveniles before seaward migration (smolt size). Indeed, large smolts typically survive better than smaller ones, particularly on bad years [65]–[67], and as our analysis illustrates, the retrospective analysis of inter-circuli spacing may reveal periods when differences among individuals become most pronounced.

An additional advantage of modeling growth based on the seasonal deposition of growth circuli is to remove bias caused by an arbitrary choice of sampling events. For example, growth of softwoods varies markedly between wood formed in spring and wood formed in summer and autumn [68], and an analysis of non-annual growth rings can provide valuable information on individual growth variation that would be lost if only annual growth rings are examined (as it is often the case in fishery science).

Accounting for individual variation in fitness traits is becoming increasingly important in ecological and evolutionary studies [69]–[72], because fitness is essentially a relative concept that needs to be interpreted in relation to performance shown by conspecifics [73]. Growth is commonly used as a proxy for fitness [74], yet comparing large number of serially correlated points, typical of growth studies, presents a considerable challenge [75]. Mixed effects modelling [76], [77] is useful in this respect, as it allows for the inclusion of random slopes and intercepts that can be used to describe the growth of individuals, as well as for the incorporation of an auto-correlation structure. Recent studies have highlighted the advantages of such an approach, and have stressed the importance of avoiding sampling individuals only twice [78], a condition common to many growth studies.

In summary, we have illustrated how a new method based on mixed-effects modelling can be used to examine individual variation in growth trajectories, reconstructed from multiple repeated measurements of the spacing between consecutive growth rings, thereby affording greater statistical power than simple growth estimates based on two or few arbitrarily chosen points in time. Fish scales can be collected non-destructively and stored dry for considerable time, providing unique archival material to address long-term population changes (e.g. [79]–[81]). Our approach makes it possible to quantify individual variation and seasonality in growth stanzas, something that popular methods such as the Von Bertalanffy growth model cannot do. The method has shown good reproducibility, and can be readily extended to the analysis of growth of other aquatic organisms having hard structures where growth rings can be visualized, such as otoliths, vertebrae, shells, or bones. We illustrate the application of the method by examining growth of migratory brown trout from two populations, based on digitized impressions of fish scales. Analysis of inter-circuli spacing during the first year of life accurately predicted whether trout migrated to sea as early (one year old) or late (2–4 year old) smolts, highlighting the profound effect that early growth can have on age at migration of anadromous fish. The fact that early migrants grew faster than older ones suggests that seaward migration was primarily triggered by ontogenetic (intrinsic) drivers, possibly in response to changes in energetic state [26], rather than by extrinsic forces such as competition with conspecifics or low food availability [24], [25].

## Supporting Information

Figure S1 **Variation in random slopes (time) and intercepts of the mixed-effects model of inter-circuli spacing during the first year of freshwater growth for a random sample of 20 sea trout from each of the two study rivers (R. Lerez, R Ulla).**
(TIFF)

Figure S2 **Coefficient of variation (CV = SD/mean) of inter-circuli spacing of freshwater growth in two populations of sea trout.** Grey bands represent point-wise 95 CI envelopes derived from bootstrapping.
(TIFF)

Figure S3 **Observed versus fitted values for the mixed-effects model of sea trout inter-circuli spacing in two populations of sea trout (R. Ulla, R. Lerez).**
(TIFF)
