# Supplementary figures and images for: Mixed-Effects Modelling of Scale Growth Profiles Predicts the Occurrence of Early and Late Fish Migrants
Source: PLoS One. 2013 Apr 16;8(4):e61744. doi: 10.1371/journal.pone.0061744 (PMC3628952; doi:10.1371/journal.pone.0061744)

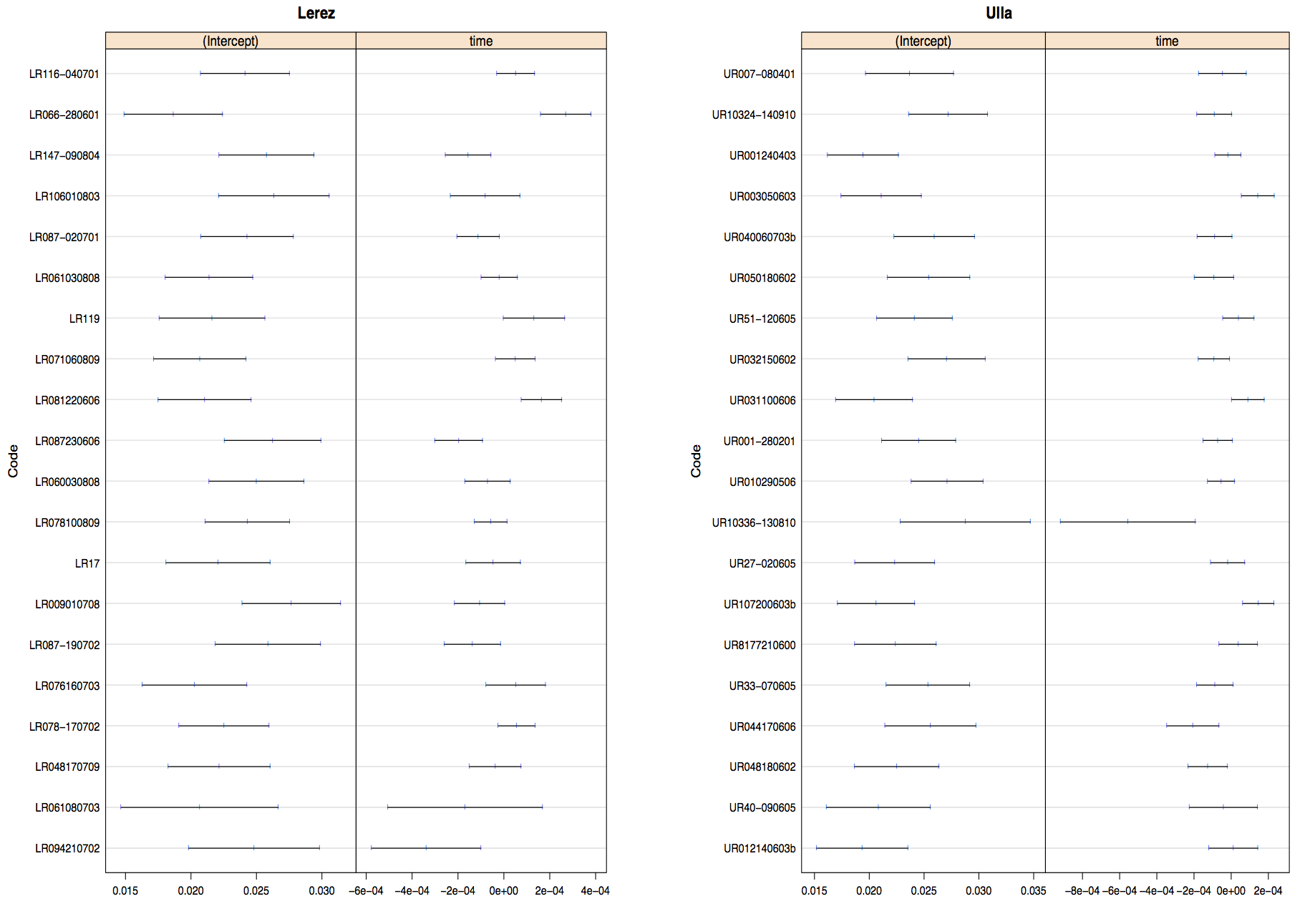

Supplement: Figure S1 — Variation in random slopes (time) and intercepts of the mixed-effects model of inter-circuli spacing during the first year of freshwater growth for a random sample of 20 sea trout from each of the two study rivers (R. Lerez, R Ulla). (TIFF) [file pone.0061744.s001.tif]

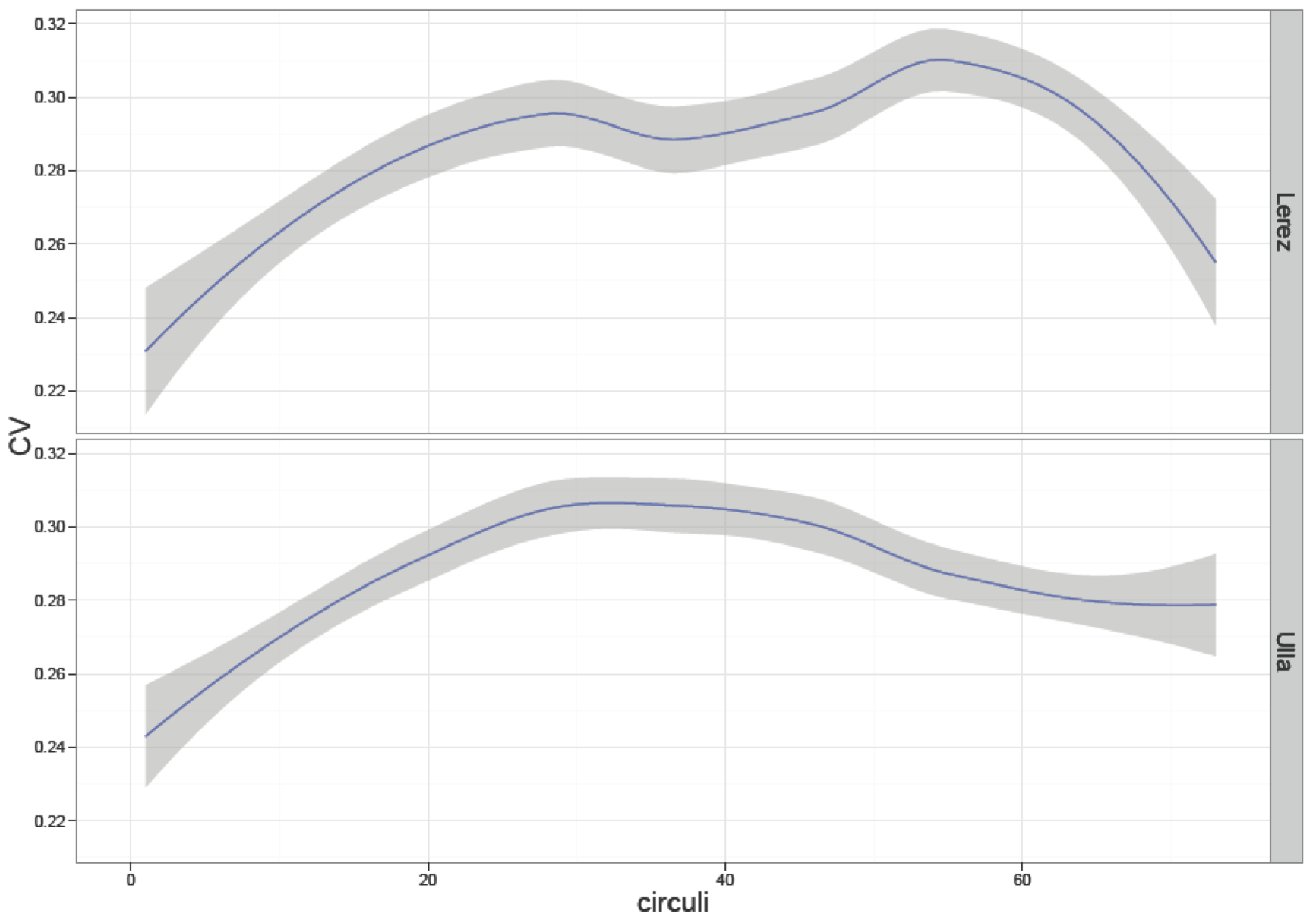

Supplement: Figure S2 — Coefficient of variation (CV = SD/mean) of inter-circuli spacing of freshwater growth in two populations of sea trout. Grey bands represent point-wise 95 CI envelopes derived from bootstrapping. (TIFF) [file pone.0061744.s002.tif]
